# A Case Report of Rare Presentation and Delay in the Diagnosis of Neuropathic Abdominal Pain Secondary to Asymptomatic Pott’s Spine in Elderly Patients

**DOI:** 10.7759/cureus.21399

**Published:** 2022-01-19

**Authors:** Bob Daripa, Arun Kumar

**Affiliations:** 1 Medicine, Grant Medical College and Sir J.J. Group of Government Hospitals, Mumbai, IND; 2 Neurology, Brahmananda Narayana Multispeciality Hospital, Jamshedpur, IND

**Keywords:** compressive fracture, skeletal tuberculosis, tuberculosis, pott’s spine, abdominal pain

## Abstract

The presence of vertebral tuberculosis (TB) in developing countries and Southeast Asia is well known, but developed nations such as the USA and UK also claim a good share because of immigrants and the HIV population. We present a unique case series of two patients with chronic abdominal pain where various differentials and arduous investigation were employed. Finally, after a few months, we could locate the lower thoracic Pott’s spine and commenced the treatment with successful resolution of symptoms.

Surgeons and gastroenterologists should rule out the spinal cause of abdominal pain and also be aware of other atypical presentations before labeling it functional or irritable bowel syndrome (IBS) or somatoform disorders. Extensive investigation, cost, delay in diagnosis, and emotional disturbances could be the end product commonly encountered in a neuropathic abdominal pain patient if a high level of suspicion is not kept at the initial presentation. Above all, potential bony deformity, neurological deficits, and their irreversible sequelae such as paraparesis can also be thwarted.

## Introduction

Sir Percivall Pott first described Potts’s spine in 1779 [1], but its evidence could be traced back about 2600 years ago around 2050 BC during the mummy era in Ancient Egypt [2]. Skeletal tuberculosis (TB) constitutes 10% of extrapulmonary TB [3], while specific spinal vertebral tuberculosis accounts for 50% of skeletal TB [1,4]. Its presence in developing countries and Southeast Asia is well known, but developed nations such as the USA and UK also claim a good share because of immigrants and the HIV population, and the incidence is on a rise [5-7]. Genetic susceptibility of the above entity has also been found recently in a Chinese population, where it is associated with vitamin D receptor gene as FokI polymorphism [6].

The common symptoms of tuberculosis are low-grade fever, back pain [4], local tenderness [6], muscular spasm [6], loss of appetite, malaise, night sweats, weight loss [4,7], and deformity [4,6], while atypical presentation could include abdominal pain, psoas or gluteal abscess, retropharyngeal abscess [6], and noncontiguous vertebral lesions [6] that can be a diagnostic dilemma and result in delayed diagnosis ranging from few days to several years [7,8], risking patient to devastating complications.

We present unique case reports of two patients with chronic abdominal pain where various differentials and arduous investigation were employed. Finally, after a few months, we could locate the lower thoracic Pott’s spine and commenced the treatment with successful resolution of symptoms.

## Case presentation

Case 1

A 65-year-old postmenopausal nondiabetic female with a past illness of chronic kidney disease following renal diet presented with abdominal pain past two months for which she had visited multiple doctors and tried many over-the-counter medications with no relief. The patient was in severe distress, lying on a bed, curved to one side, holding her belly. She narrated the pain as insidious onset, generalized, continuous, deep, dull aching. The pain was more pronounced over the right hypochondriac region, not related to breathing, with no aggravating or relieving factors. The patient was not able to do routine household duties because of the pain and therefore acquired a low mood. There was no nausea, vomiting, loose stool, or bladder complaint. Instead, she does have occasional constipation for which she relies on home remedies. There are no constitutional symptoms of fever, lack of appetite, or weight loss, and the pain does not disturb sleep. There was no back pain or black stool. The patient never tried alcohol or any recreational drugs and denies any accident or injury.

On examination, the abdomen was soft with minimal generalized tenderness. There was no rigidity or rebound tenderness, and no organomegaly or lymphadenopathy. The lungs were clear on auscultation, and heart sounds were normal. Carnett’s sign was negative. There was no spine tenderness. Limb tone and reflex were normal, with equivocal planters. Blood and urine workup were within normal limits. The ESR was 42 mm in the first hour. The creatinine level was 1.5 mg/dL, which is her baseline. Multiple abdominal roentgenograms and ultrasounds were done in the last two months with normal findings. The patient was treated with antispasmodic and antihelminthic with no relief. CT scan of the whole abdomen and pelvis was unremarkable. Barium series studies, upper GI endoscopy, colonoscopy, invasive gynecological procedures, and all possibilities of exploratory laparotomy were discussed by surgeons and gastroenterologist. Unfortunately, the patient was lost to follow-up for unknown reasons.

A few months later, the patient showed up again with unbearable dull aching abdominal pain. The pain has been there on daily basis, almost continuous, and associated with a significant weight loss. There was a sense of tightness around the waist with lower limb weakness and difficulty walking with easy fatiguability, and the patient, therefore, needed a stick and support of one person to ambulate for the last few weeks. Also noted was occasional tingling paresthesia of both lower limbs. Constipation has become more pronounced but not back pain, bone pain, or black stool. There was no recent heavy weight lifting or ataxic gait leading to falls.

**Figure 1:**
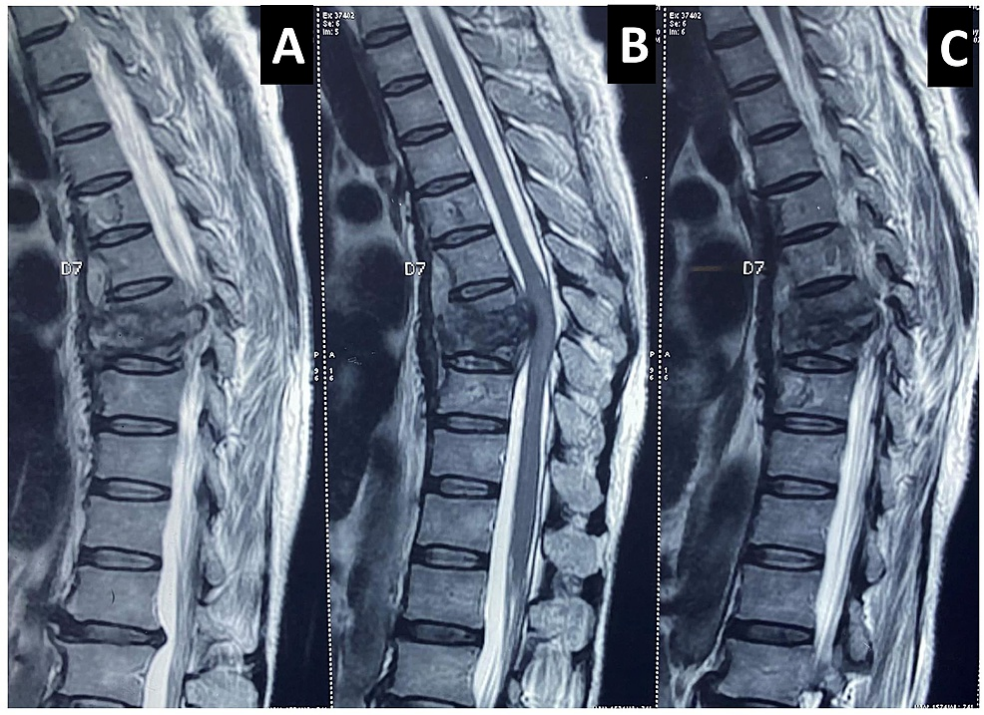
A, B, and C: Sagittal view T2-weighted MRI of the thoracic spine showing D8 vertebral body bony lesion, obliterated disc margins with anterior wedge necrosis. The vertebral body shows extensive tuberculous destruction bulging into the spinal cord with angulation of the bony column. D6, D7, D8, and D9 have central vertebral body lesions where the anterior longitudinal ligaments also look hypertrophied. The spinal cord at the D8 region looks compressed, but no edema or signal intensity changes were noted. There is no pre- or paravertebral cold abscess or gibbus formation. Discs, pedicles, and lamina are well preserved.

On examination, there was mild lower limb muscle wasting but no visible spine deformity or kyphosis. There was no spine tenderness, and the tone in both lower limbs seemed slightly raised with exacerbated knee jerks bilaterally but hypoactive ankle reflex. Planters were equivocal. Power in both lower limbs was 4/5, but the upper limbs have full strength. The sensation was intact to light touch and pinprick with no sensory level. The rectal tone was intact. The lungs and cardiac examination were normal. Her ESR levels were remarkably raised, and other blood and urine parameters are shown in Table 1. Thoracic MRI scan surprisingly revealed D8 vertebral body bony lesion suggestive of spinal TB, as shown in Figure 1. QuantiFERON-TB also reported high positivity. Sputum is negative for acid-fast bacilli (AFB), and chest X-ray is unremarkable.

**Table 1:** Demographic and relevant laboratory values of case 1 and case 2. GOD-POD: glucose oxidase-peroxidase; R&M: routine and microscopy

	Case 1	Case 2	Normal reference range
Age (years)	65	76	-
Sex	Female	Male	-
Body weight (kg)	52	55	-
Comorbidity	Chronic kidney disease	Nil	-
Hemoglobin (Hb) (gm/dL)	9.6 (low)	10.3 (low)	12–15
MCV (fL)	66 (low)	72 (low)	83–101
Peripheral blood smear	Hypochromic microcytic	Hypochromic microcytic	-
Total leucocyte count (TLC) (cells/mm^2^)	4,100	7,900	4,000–10,000
ESR (modified Westergren method) (mm)	62 (raised)	67 (raised)	0–15 (at the end of one hour)
CRP (mg/L)	2.8 (normal)	6.5 (normal)	<10 (immunoenzymatic method)
Fasting blood glucose (mg/dL)	92	78	<100 (GOD-POD method)
Creatinine (mg/dL)	2.1 (baseline: 2.0)	1.3	0.66–1.25
BUN (mg/dL)	14.3 (normal)	11.9 (normal)	9–20
AST (U/L)	41	35	17–59
ALT (U/L)	33	40	<50
Total proteins (mg/dL)	6.5	7.2	6.3–8.2
Calcium (mg/dL)	8.1 (low)	7.9 (low)	8.4–10.2
Phosphorous (mg/dL)	Normal	2.3 (low)	2.5–4.5
Alkaline phosphatase (U/L)	97	118	38–126
1,25-Dihydroxycholecalciferol (vitamin D) (ng/mL)	Low	Low	Insufficiency < 30
Protein electrophoresis	Normal	Normal	-
Urine Bence–Jones protein	Negative	Negative	-
Urine R&M	Normal	Normal	-
Stool examination	Normal	Normal	-
HIV 1 and 2	Negative	Negative	ELISA method
Sputum for AFB stain	Negative	Negative	-
QuantiFERON-TB Gold (gamma interferon for TB)	Positive	Positive	ELISA method
Sputum for AFB stain	Negative	Negative	-
Chest X-ray	No infective foci	No infective foci	-
Whole abdomen USG	Unremarkable (no calculi, no mass, no organomegaly, no lymphadenopathy)	Unremarkable (no calculi, no mass, no organomegaly, no lymphadenopathy)	-

Diagnosis of D8 Pott’s spine with atypical abdominal pain presentation was made. Antitubercular therapy was started (isoniazid 5 mg/kg, rifampicin 10 mg/kg, pyrazinamide 25 mg/kg, ethambutol 15 mg/kg with pyridoxine 20 mg) with conservative management. After two months of follow-up, a gradual improvement in abdominal symptoms was noted, and she was able to ambulate herself without any limb tightness. The antitubercular dose was reduced to isoniazid and rifampicin and continued for the next 10 months. On recent follow-up after 1.5 years, the patient is pain-free. Informed written consent was obtained from the patient for the publication of this case report.

Case 2

Recently, a 76-year-old elderly hypertensive male with no other comorbidities presented with bilateral flank and abdominal pain for the past two months. The pain was dull with acute onset almost daily, more pronounced in the flanks and bilateral hypochondriac region, noted when rising from sitting or squatting position or walking. This disturbing pain stays for around an hour before it resolves on its own or needs occasional oral intake of tramadol or paracetamol for symptomatic relief as prescribed by surgeons. There was no back pain, bowel and bladder complaint, black stool, or any limb weakness or sensory deficit. Constitutional symptoms were not present, and the patient sleeps well supine. Two years back, there was a history of falls from the pillion seat of the bike due to bike skid. Post fall, there were no emergency hospital visits or major injury or fracture of any kind. The patient is fully vaccinated for COVID-19 and is nonalcoholic and nonsmoker.

On examination, the abdomen was soft with no tenderness or palpable organomegaly. Rectal examination revealed normal rectal tone and prostate. Cardiac and respiratory examinations were unremarkable. Carnett’s sign was negative. There was no neurodeficit. All limb’s tone and power were adequate. Sensory examination was normal to pinprick and touch. Reflexes were normal in all limbs. Planters were downgoing. Mild lower thoracic vertebral tenderness was noted but no gibbus or deformity.

**Figure 2:**
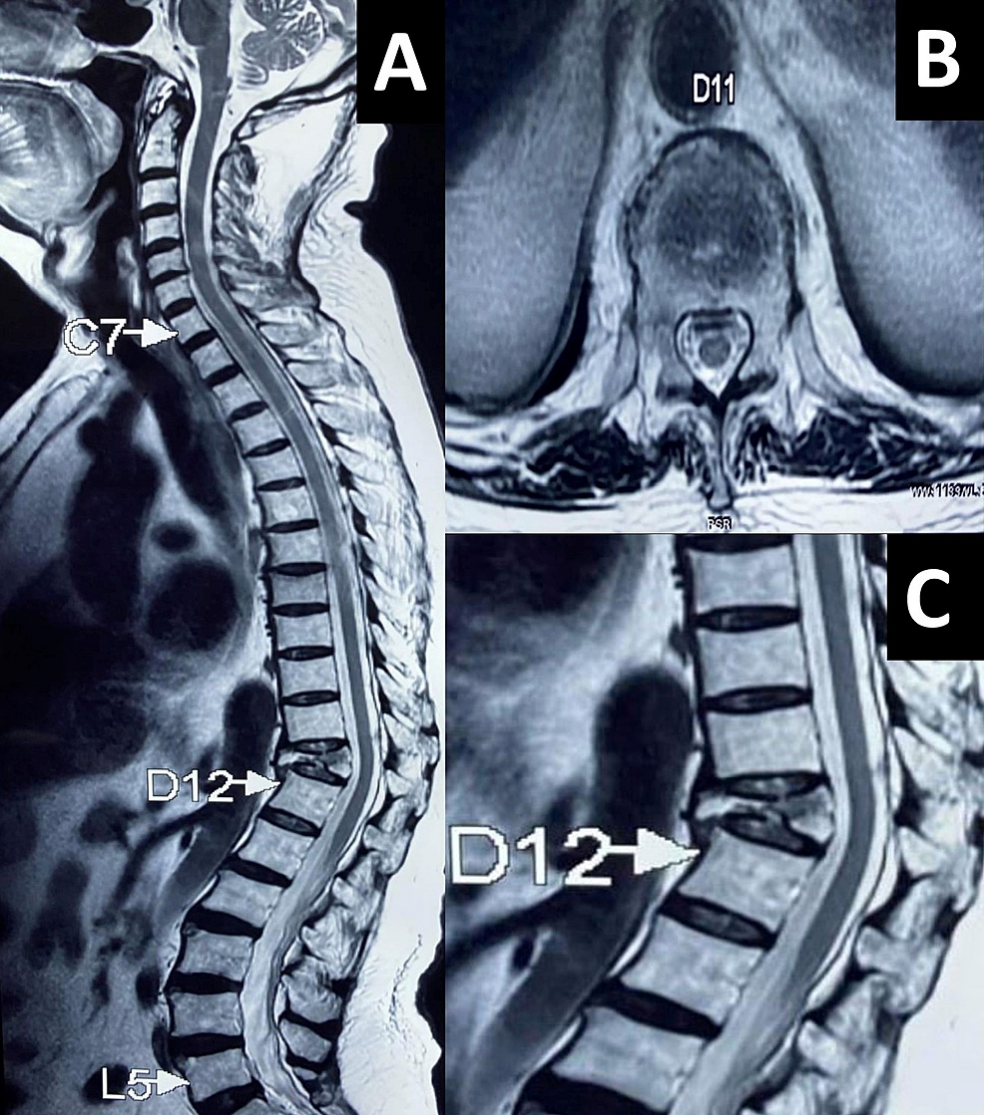
A: T2-weighted longitudinal cross-sectional image of the whole spine with a thoracic D11 vertebral body extensive bony necrotic lesion with complete tuberculous destruction of the body and obliterated disc margins. Also noted was a small grade I compression of the thoracic D7 vertebral body. B: Axial cross-sectional image at thoracic D11 level showing a small posterior epidural retropulsed component just intending the thecal sac. C: Grade III compression fracture of the D11 vertebral body with hypertrophied anterior longitudinal ligaments, but the spinal cord looks spared with no edema or signal intensity changes. Pre- or paravertebral cold abscess or gibbus formation not seen. The discs, pedicles, and lamina also seem to be intact.

Blood investigation and other diagnostic parameters are shown in Table 1, along with case 1. The patient’s ESR levels were very high. Abdominal and chest roentgenogram and whole abdominal ultrasound were normal with no calculi. X-ray of the thoracolumbar region revealed compressed D11 thoracic vertebrae. MRI screening of the whole spine reported D7 and D11 compressive vertebral fracture with mild spinal cord compression/spinal root compression, as shown in Figure 2. QuantiFERON-TB exhibited very high positivity suggestive of tuberculosis infection. Sputum tested negative for acid-fast bacilli with an unremarkable chest X-ray.

Conservative management with antitubercular and oral GABAnergic medications was started immediately based on body weight (isoniazid 5 mg/kg, rifampicin 10 mg/kg, pyrazinamide 25 mg/kg, and ethambutol 15 mg/kg with pyridoxine 20 mg). After two weeks, the patient claimed that the flank and abdominal pain were better. Informed written consent was given by the patient for the publication of this report.

## Discussion

**Figure 3:**
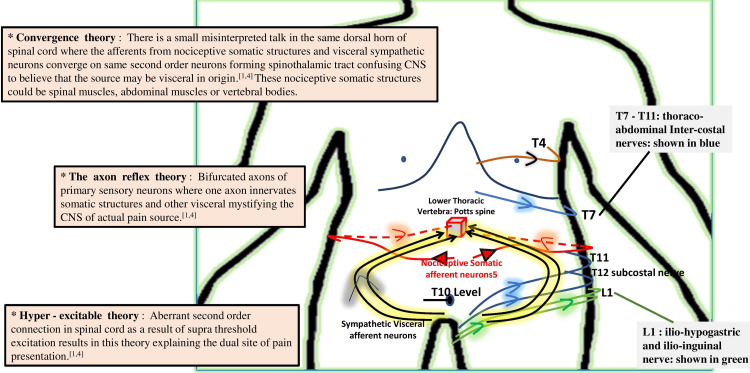
A simplified representation of neuropathic abdominal pain due to Potts’s spine at thoracic vertebrae. Black color curved lines represent the visceral afferent nerves, and red color lines represent the nociceptive somatic afferent nerves converging on second-order neurons, forming the spinothalamic tract. Blue and green lines represent the anterior cutaneous nerves of the abdominal wall. Three theories explaining the pathomechanism are shown in pink color boxes on the left side (marked *).

Only a handful of such atypical presentations of spinal tuberculosis cases are reported so far [3,5]. Our cases are unique as it generates awareness of the abdominal presentation of spinal TB despite no abdominal pathology. Although it is worth mentioning here that in case 1, the CT scan did report about the normal abdominal anatomy but missed out on thoracic vertebrae findings. Spinal TB is usually seen in the lower thoracic and upper lumbar vertebrae [1,4]. Studies have shown that thoracic level spinal cord injury bestowed cramping, tightening, or dull character abdominal discomfort with constipation, significantly affecting the quality of life [9], and interestingly, even parietal hemorrhagic infarction has witnessed acute abdominal presentation in emergency [10]. Various theories explaining the pathomechanisms were proposed in the past, namely, convergence theory, axon reflex theory, and hyper-excitable theory [1,4], which are summarized in Figure 3 in pink boxes.

In spinal TB, the route of entry could be distant foci such as the gut, kidney, tonsils, para-aortic lymph nodes, hematogenous seeding, or Batson’s venous plexus [4]. Para-diskal lesions, anterior wedging, or collapse are commonly seen because of the arterial distribution pattern, while venous plexus causes more central lesions in the vertebral bodies [6]. Noncontagious vertebral involvement could be due to posterior ligamentous spread [6]. All these features could be easily picked up in MRI, which is considered the best diagnostic modality, undoubtedly more sensitive and specific than roentgenography and CT scan, respectively [7]. Few drawbacks in our case reports are reported in Table 2.

**Table 2:** Limitations noted in our case reports.

All modalities mentioned below can have false-negative results, so it is advisable to diagnose spine TB based on clinical acumen aided with radiological evidence [6].
No biopsy or FNAC for culture or ZN stain was done from the concerned pathological site of spine lesion as their results vary [6,7].
Bronchoalveolar lavage or bronchial wash was not tested as the patient did not have any respiratory symptoms, and the chest X-ray was also normal.
CT-guided FNAC of the infective foci could prove to be an important diagnostic tool, which was not done here, as many times it cannot isolate the bacilli [7].
Roentgenogram may show a vertebral fracture, believing it to be compressive fracture and delaying TB spine diagnosis as evidenced earlier [4], so it was not done here.
PPD skin test was not done as it is nonspecific and could be false positive or false negative [7].
Surgical exploration was not done here, which has a role in abscess debridement and spinal stabilization [3,6], although its need and benefits are controversial [3].
A bone scan can differentiate metastatic lesions, but 63% of patients with Pott’s spine can have similar uptake of technetium 99m[6], so it has limited and doubtful utility.

## Conclusions

It is important to note that timely diagnosis of such atypical presentation can avoid potential bony deformity, neurological deficits, and irreversible sequelae such as paraparesis. Surgeons and gastroenterologists should rule out the spinal causes of abdominal pain before labeling it functional or irritable bowel syndrome (IBS) or somatoform disorders. We are fortunate that the onset of symptoms in such cases is usually insidious and progresses slowly, so we have time to work up in detail for a diagnosis. However, in doing so, it exposes the patients to a variety of investigations, procedures, scans, and even surgeries. Radiological mimickers of spinal TB such as malignancy or compressive fractures could further confuse the diagnosis.
